# Impact of a 12-week obesity intervention on menopausal symptoms and psychological well-being across menopause stages: a cross-sectional analysis

**DOI:** 10.3389/frph.2025.1524790

**Published:** 2025-05-21

**Authors:** Rupal Kumar, Moattar Raza Rizvi, Waqas Sami

**Affiliations:** ^1^ Department of Nutrition and Dietetics, School of Allied Health Sciences, Manav Rachna International Institute of Research and Studies, Faridabad, India; ^2^ College of Healthcare Professions (CoHP), D.I.T University, Dehradun, India; ^3^ Department of Pre-Clinical Affairs, College of Nursing, QU Health Sector, Qatar University, Doha, Qatar

**Keywords:** menopause, obesity, body composition, psychological well-being, obesity interventions, menopausal rating scale 2.2 sample size calculation

## Abstract

**Introduction:**

Menopause poses unique physical and psychological challenges, especially for obese women, impacting quality of life across menopausal stages. This study evaluates a 12-week obesity educator program on body composition, menopausal symptoms, and psychological well-being among pre-, peri-, and post-menopausal obese women in Delhi NCR, India.

**Methods:**

A cross-sectional analysis was conducted with 291 obese women (97 per menopausal stage) assessing anthropometric measures, menopausal symptoms via the Menopause Rating Scale (MRS), and psychological well-being using the Well-being Questionnaire (W-BQ12). Data were analyzed using paired t-tests, ANCOVA, Pearson's correlation, and regression analysis.

**Results:**

The intervention led to significant weight reduction across all groups (3.8–4.5 kg, *p* < 0.001), waist circumference decreases (5.7–6.5 cm, *p* < 0.001), and HbA1c reductions by 0.4% (*p* < 0.001). Regression analysis identified waist-to-hip ratio and hemoglobin as predictors of somato-vegetative and psychological symptoms (*R*^2^ = 0.15, *p* < 0.05). MRS scores showed the most improvement in perimenopausal women.

**Conclusion:**

The obesity educator program effectively improved body composition, glycemic control, and well-being across menopausal stages, highlighting the value of personalized interventions for menopausal health management.

## 1 Introduction

Menopause is a pivotal phase in a woman's life, marking the cessation of ovarian function and the end of reproductive capacity. Typically occurring between the ages of 45 and 55, it is characterized by a significant decline in estrogen and progesterone levels, which leads to a variety of physical, psychological, and emotional changes. As life expectancy increases globally, women now spend a substantial portion of their lives in the post-menopausal phase, which underscores the importance of managing the health challenges associated with menopause. In India, menopause tends to occur earlier than the global average, with women transitioning as early as 45 years. This earlier onset, combined with cultural factors that affect how symptoms are perceived and managed, creates a unique context for studying menopausal experiences in Indian women (1).

The menopausal transition can be broken down into three distinct stages: premenopause, perimenopause, and postmenopause. During premenopause, menstrual cycles are regular, and hormone levels fluctuate within normal ranges. However, as women enter perimenopause, they begin to experience irregular menstrual cycles, and fluctuations in hormone levels become more pronounced, leading to a variety of symptoms such as hot flashes, mood swings, sleep disturbances, and weight gain (2). These symptoms often intensify during perimenopause, peaking in both frequency and severity. Once a woman has gone 12 consecutive months without menstruating, she is considered postmenopausal. Although some symptoms, such as hot flashes, may subside during this stage, postmenopausal women face increased risks of osteoporosis and cardiovascular diseases due to sustained low estrogen levels (3).

Globally, menopausal symptomatology has been widely studied, especially in Western populations, where the experiences of women have been well-documented. However, the experiences of Indian women during menopause remain under-researched, with many studies failing to account for cultural differences in symptom reporting and management. In India, menopause is still considered a taboo subject, with societal norms often discouraging open discussions about the physical and emotional challenges that accompany it (4). Recent evidence highlights that only 18.1% of rural Indian women are aware of menopausal symptoms, compared to 51.4% of their urban counterparts, with minimal awareness about treatment options like hormone therapy (5). Similarly, a cross-sectional study from Puducherry reported a 96.6% prevalence of menopausal symptoms, with physical complaints such as fatigue and joint pain being the most common (6), while another from Haryana found that 87.7% of women experienced significant quality-of-life impacts due to menopausal symptoms (7). Cultural taboos, religious beliefs, and variations in education and geographic location further influence symptom recognition and reporting behaviors, limiting timely access to care. Additionally, Indian women often experience menopause earlier than their Western counterparts and may have different symptom profiles, with musculoskeletal pain and psychological issues like anxiety and depression being more prominent (1). Despite the physical and emotional toll that menopause can take, there remains a gap in understanding how demographic factors, such as age, education, body mass index (BMI), and lifestyle, influence the severity of these symptoms and overall well-being.

A major area of interest in menopause research is the relationship between symptom severity and well-being. The Menopause Rating Scale (MRS) and Well-being Questionnaire (W-BQ12) are widely used to measure the intensity of menopausal symptoms and psychological well-being, respectively. However, these tools have been underutilized in studies focusing on Indian populations, as most research on menopausal health in India has predominantly employed general symptom checklists rather than validated instruments like the MRS and W-BQ12 (1, 4, 8). Given the demographic differences between Indian and Western women, there is a pressing need to investigate how factors like age, BMI, education, and lifestyle habits affect both the severity of menopausal symptoms and overall psychosocial well-being in Indian women. For instance, it is hypothesized that women in perimenopause will report the highest severity of symptoms compared to those in the pre- and post-menopausal stages, given the intense hormonal fluctuations during this period. Similarly, demographic factors such as higher education levels and healthier lifestyle habits may correlate with improved well-being and lower symptom severity across all stages of menopause (9).

However, these tools have been underutilized in studies focusing on Indian populations, as most research on menopausal health in India has predominantly employed general symptom checklists rather than validated instruments like the Menopause Rating Scale (MRS) and W-BQ12 (1, 4, 8).

Previous studies have demonstrated that obesity significantly influences the severity of menopausal symptoms and psychological well-being. Obese women tend to experience more frequent and severe vasomotor and somatic complaints, including hot flashes, fatigue, and joint pain (10, 11). Central obesity has also been associated with increased emotional distress and depressive symptoms during menopause (12). Furthermore, lifestyle interventions that promote weight loss through dietary modifications and physical activity have shown improvements in both physical and mental health outcomes among menopausal women (13–15). These findings underscore the need for holistic, personalized approaches that address both physiological and psychological dimensions of menopausal health. Furthermore, recent evidence emphasizes that diet plays a central role in managing weight gain and metabolic disturbances during menopause, reinforcing the relevance of nutrition-focused interventions in midlife women (16).

This study aims to address critical gaps in understanding menopausal health among Indian women by examining how a 12-week obesity educator program affects body composition, menopausal symptoms, and well-being across menopausal stages. Using validated tools such as the MRS and W-BQ12, it investigates how demographic factors such as age, education, body composition, and lifestyle affect symptom severity and well-being across menopausal stages. The findings aim to enhance culturally sensitive healthcare practices that improve the quality of life for menopausal women in India. The findings are intended to inform the development of personalized culturally sensitive interventions that improve both physical and mental health outcomes, guiding healthcare providers in delivering holistic care for menopausal women in India and contributing to broader global strategies as the postmenopausal population continues to grow.

## 2 Materials and methods

### 2.1 Study design

This study utilized a quasi-experimental, longitudinal research design to evaluate the impact of a 12-week obesity educator program on menopausal symptoms, well-being, and demographic variables among obese women in the Delhi National Capital Region (NCR), India. The study was conducted across three menopausal stages—pre-menopause, peri-menopause, and post-menopause—to analyze how symptom severity and well-being differ based on demographic factors like age, body composition, and education.

### 2.2 Sample size calculation

The sample size for this study was calculated using G-Power, yielding a required sample size of 289 participants. This calculation was based on a confidence level of 95% (alpha = 0.05) and an expected effect size of 0.75, as suggested (17). To ensure the accuracy of the sample size estimate, we incorporated the finite population correction factor, which is crucial when the total population size (*N*) is not significantly larger than the sample size (*n*). The correction reduces bias when the ratio of *n* to *N* is close. The formula used for this adjustment was:

$$n=\frac{N\;\times \;Z^{2}\;\times \;p\;\times \;q}{d^{2}\;\times \;(N-1)+Z^{2}\;\times \;p\;\times \;q}$$

where *n* is the adjusted sample size, N is the total population size, Z is the *Z*-score for the desired confidence level, *p* is the estimated proportion, (q = 1-p) and d is the margin of error. The finite population correction factor provided a more precise sample size estimate. However, as the ratio *n*/*N* was less than 0.05, the finite population correction was disregarded in the final calculation, streamlining the process.

### 2.3 Participants and sampling

A total of 291 obese women, aged 18–60 years, were recruited using a stratified random sampling method to ensure equal representation across the three menopausal stages: pre-menopausal (97 women), peri-menopausal (97 women), and post-menopausal (97 women). Participants had to meet the inclusion criteria, which required them to have a BMI ≥ 25 kg/m^2^ (according to the Asian-Pacific classification) and be willing to participate in a 12-week lifestyle intervention program. The study included women aged 45–65 years residing in the Delhi NCR region, who were randomly selected using stratified sampling methods from community clinics and health centers to ensure equal representation across menopausal stages. Inclusion criteria were: (a) BMI greater than 25 kg/m^2^; (b) currently experiencing menopause symptoms; (c) confirmed menopausal stage based on the World Health Organization (WHO) criteria. Exclusion criteria included any chronic illnesses, use of hormone replacement therapy, and history of surgical menopause. Additionally, women with eating disorders, renal or hepatic diseases, or smoking habits were also excluded from the study. Participants were stratified by menopausal stage (premenopausal, perimenopausal, and postmenopausal), verified through medical records and self-reported menstrual history.

### 2.4 Intervention

The study implemented a structured 12-week obesity educator program as an intervention, aimed at improving body composition and alleviating menopausal symptoms through lifestyle modifications. The program included biweekly group sessions led by certified educators, each lasting approximately 90 min. Sessions covered balanced nutrition, physical activity, and stress management techniques tailored for menopausal women. As part of the nutritional education, participants were guided to follow a dietary plan providing approximately 1,200–1,500 kcal/day, adjusted based on individual baseline body weight (approximately 20–25 kcal/kg/day). The macronutrient distribution was standardized to 50%–55% carbohydrates, 20%–25% protein, and 25%–30% fats, emphasizing low-glycemic index foods, lean proteins, and healthy fats. These dietary plans were personalized and reviewed weekly to ensure adherence. Educators adhered to a standardized curriculum to ensure uniform content delivery. Participant adherence was tracked through session attendance and self-reported logs of lifestyle changes made during the program. Data was collected using validated tools and objective measurements at baseline (0 week) and post-intervention (week 12).

### 2.5 Data collection instruments

#### 2.5.1 Anthropometry and body composition

Anthropometric assessments were conducted at both baseline and post-intervention to measure changes in body composition. The following parameters were assessed:

##### 2.5.1.1 Height and weight

Height was measured using a stadiometer (SECA 220, Seca Corporation, Hamburg, Germany) with participants in a standing posture without shoes, recorded to the nearest 0.5 cm. Weight was measured using a calibrated Omron Karada Scan Body (model HBF-375), with participants in minimal clothing and without shoes, recorded to the nearest 0.1 kg. Body Mass Index (BMI) was calculated as weight (kg) divided by height squared (m^2^).

##### 2.5.1.2 Waist and hip circumference

Waist circumference (WC) was measured at the iliac crest using a Gulick tape measure, and hip circumference was measured at the widest part above the gluteal fold, both rounded to the nearest 0.25 cm. The waist-to-hip ratio (WHR) was calculated to assess abdominal obesity.

#### 2.5.2 Fat mass (FM) and skeletal muscle mass (SMM)

Body composition, including fat mass and skeletal muscle mass, was measured using a bioelectrical impedance analysis (BIA) device (Omron, Karada model HBF-375). The assessments were performed in the morning after a 12-h fast. This device, portable and reliable, provided estimates of body fat percentage and muscle mass. For safety reasons, individuals with pacemakers were excluded from this assessment, as the device emits a small electrical current during measurements

#### 2.5.3 Menopause rating scale (MRS)

The MRS was employed to assess the severity of vasomotor, somatic, psychological, and urogenital symptoms. Each symptom was rated on a scale from 0 (no symptoms) to 4 (very severe symptoms), allowing for a comprehensive evaluation of menopausal symptoms across the three stages at both baseline and post-intervention. A Hindi-translated and validated version of the MRS (MRS-H) has been previously developed and tested among Indian women (8). Moreover, the scale has been successfully applied in Indian populations in both northern (18) and southern regions (19), supporting its contextual relevance. The reliability and internal consistency of the MRS has also been demonstrated in global validation studies (20).

#### 2.5.4 Well-being questionnaire (W-BQ12)

The W-BQ12 was used to assess psychological well-being, including general well-being (GWB), positive well-being (PWB), negative well-being (NWB), and energy levels (ENE). This tool included 12 items that provided insights into how psychological well-being was influenced across different menopausal stages, with items scored on a 4-point Likert scale ranging from 0 (not at all) to 3 (all the time), where higher scores indicated better overall well-being. The W-BQ12 has previously been applied in Indian clinical settings (21, 22), and though a formally adapted Indian version is not yet validated, its use has been supported. The reliability of the tool has also been confirmed in one of the Korean study (23). The internal consistency (Cronbach's alpha) for this study is presented in the results section.

### 2.6 Ethical approval

The study was approved by the Ethical Committee at the Faculty of Allied Health Sciences (Reference No. MRIIRS/FAHS/March/2022/M-007 dated 2nd March 2022) and adhered to the Ethical Principles for Medical Research involving human subjects as outlined in the WMA Declaration of Helsinki. Written informed consent was obtained from all participants prior to their inclusion in the study. Participants were assured of the confidentiality of their data, and the study was conducted ensuring the privacy and anonymity of all individuals. Additionally, participants were informed of their right to withdraw from the study at any time without any repercussions. The study followed all institutional guidelines for ethical research practices.

### 2.7 Data analysis

Data analysis was performed using SPSS version 23.0. Descriptive statistics were employed to summarize the demographic characteristics, menopausal symptoms, and well-being scores. Analysis of Covariance (ANCOVA) was used to evaluate the effects of demographic factors (e.g., age, BMI, education) on menopausal symptoms and well-being. Changes in symptom severity and well-being before and after the intervention were assessed using paired t-tests, while Pearson's correlation was employed to examine the relationships between demographic variables and menopausal symptoms. Effect sizes (Cohen's *d*) were provided for significant findings to quantify the magnitude of observed effects.

## 3 Results

A total of 291 participants were stratified equally into premenopausal, perimenopausal, and postmenopausal groups (*n* = 97 each). One-way ANOVA revealed a statistically significant difference in mean age across the three groups (*F* = 241.35, *p* < 0.001), consistent with their classification by menopausal stage. Chi-square analyses demonstrated significant associations for marital status (*χ*^2^ = 13.87, *p* = 0.03) and age at marriage (*χ*^2^ = 7.24, *p* = 0.03). Notably, a higher proportion of widowed women was observed in the postmenopausal group, likely reflecting cumulative age-related spousal loss. Early marriage (<20 years) was also more prevalent among postmenopausal women, suggesting generational differences in reproductive timing. No significant group differences were observed for education level (*χ*^2^ = 7.54, *p* = 0.27), age at menarche (*χ*^2^ = 0.56, *p* = 0.76), pregnancy history (*χ*^2^ = 3.06, *p* = 0.22), smoking status (*χ*^2^ = 1.17, *p* = 0.56), or number of births (*χ*^2^ = 8.17, *p* = 0.09). These findings suggest that while certain socio-reproductive factors like marital history and age at marriage vary significantly across menopausal stages, others such as education and parity remain relatively stable across groups in this population (Table 1).

**Table 1:** Distribution of demographic and reproductive variables across menopausal stages.

Variable	Premenopause (*n* = 97)	Perimenopause (*n* = 97)	Postmenopause (*n* = 97)	*χ* ^2^	***p***-value
Age (Mean ± SD)	37.91 ± 4.49	42.31 ± 5.24	53.32 ± 5.72	241.4	*p* < 0.001
Education					
No formal education	8 (8.2%)	12 (12.4%)	17 (17.5%)	7.54	0.27
Basic education	25 (25.8%)	28 (28.9%)	32 (33.0%)		
Secondary education	38 (39.2%)	37 (38.1%)	33 (34.0%)		
Higher education	26 (26.8%)	20 (20.6%)	15 (15.5%)		
Marital status					
Married	91 (93.8%)	85 (87.6%)	81 (83.5%)	13.87	0.03*
Unmarried	5 (5.2%)	7 (7.2%)	4 (4.1%)		
Widow	0 (0%)	4 (4.1%)	11 (11.3%)		
Separated	1 (1.0%)	1 (1.0%)	1 (1.0%)		
Age at menarche					
<15 years	64 (66.0%)	61 (62.9%)	59 (60.8%)	0.56	0.76
≥15 years	33 (34.0%)	36 (37.1%)	38 (39.2%)		
Age at marriage					
<20 years	53 (54.6%)	62 (63.9%)	71 (73.2%)	7.24	0.03*
≥20 years	44 (45.4%)	35 (36.1%)	26 (26.8%)		
Ever been pregnant					
Yes	90 (92.8%)	93 (95.9%)	95 (97.9%)	3.06	0.22
No	7 (7.2%)	4 (4.1%)	2 (2.1%)		
Smoking status					
Never smoked	94 (96.9%)	93 (95.9%)	91 (93.8%)	1.17	0.56
Current smoker	3 (3.1%)	4 (4.1%)	6 (6.2%)		
Number of births					
None	6 (6.2%)	3 (3.1%)	1 (1.0%)	8.17	0.09
One	15 (15.5%)	14 (14.4%)	12 (12.4%)		
Two	47 (48.5%)	45 (46.4%)	39 (40.2%)		
>2	29 (29.9%)	35 (36.1%)	45 (46.4%)		

*χ*^2^ = Chi-square test statistic. *p < 0.05.

The effects of the 12-week obesity educator program were evaluated through Wilcoxon signed-rank tests and paired *t*-tests across premenopause, perimenopause, and postmenopause groups. Significant reductions in weight were observed in all groups (Table 2): premenopause (*Z* = 0, *p* < 0.001), perimenopause (*Z* = 0, *p* < 0.001), and postmenopause (*Z* = 0, *p* < 0.001), with large effect sizes. Similarly, BMI significantly decreased across all groups. Waist Circumference (WC) and Hip Circumference (HC) showed significant reductions in all groups, while Waist-to-Hip Ratio (WHR) improved with small effect sizes. In terms of biochemical parameters, HbA1C and Glucose levels showed significant reductions across all groups (*p* < 0.001), indicating improved glycemic control. Hemoglobin significantly increased in the perimenopause group (*Z* = 132, *p* < 0.001), with no significant changes in the other groups. For physical parameters, Systolic Blood Pressure (SBP) significantly increased in the premenopause group (*Z* = 1,064, *p* < 0.001), while Diastolic Blood Pressure (DBP) significantly decreased in both the premenopause and postmenopause groups (*p* < 0.001). Skeletal Muscle Mass (SMM) significantly increased across all groups, and Fat Mass showed significant decreases, reflecting positive changes in body composition.

**Table 2:** Comparison of anthropometric, biochemical, physical, and body composition parameters across menopausal stages before and after the 12-week obesity educator program.

Anthropometry							
Outcome measure	Group	Pre (Mean ± SD)	Post (Mean ± SD)	Test	Statistic	*p*-value	Effect size-***d***/***r***
Height	Premenopause	1.55 ± 0.11	1.54 ± 0.10	Wilcoxon	0	<0.001	0
	Perimenopause	1.51 ± 0.15	1.52 ± 0.12	Wilcoxon	376	0.63	0.08
	Postmenopause	1.51 ± 0.14	1.50 ± 0.11	Wilcoxon	176	<0.01	0.04
Weight	Premenopause	69.44 ± 3.82	63.31 ± 5.56	Wilcoxon	0	<0.001	0
	Perimenopause	71.60 ± 5.03	67.40 ± 5.27	Wilcoxon	0	<0.001	0
	Postmenopause	73.30 ± 5.56	66.80 ± 5.98	Wilcoxon	0	<0.001	0
BMI	Premenopause	29.24 ± 4.23	27.13 ± 3.95	Wilcoxon	203	<0.001	0.04
	Perimenopause	32.05 ± 6.25	30.28 ± 4.62	Wilcoxon	639	<0.001	0.13
	Postmenopause	33.77 ± 6.42	30.07 ± 5.13	Wilcoxon	0	<0.001	0
WC	Premenopause	77.96 ± 5.54	73.31 ± 4.65	Wilcoxon	0	<0.001	0
	Perimenopause	77.35 ± 6.23	69.05 ± 5.32	Wilcoxon	0	<0.001	0
	Postmenopause	83.02 ± 6.74	71.92 ± 7.34	Wilcoxon	0	<0.001	0
HC	Premenopause	95.07 ± 10.80	92.32 ± 10.49	Wilcoxon	0	<0.001	0
	Perimenopause	90.50 ± 5.61	87.22 ± 5.45	Wilcoxon	0	<0.001	0
	Postmenopause	92.81 ± 9.04	85.62 ± 8.48	Wilcoxon	0	<0.001	0
WHR	Premenopause	0.83 ± 0.10	0.80 ± 0.10	Wilcoxon	70	<0.001	0.01
	Perimenopause	0.86 ± 0.09	0.79 ± 0.08	Wilcoxon	0	<0.001	0
	Postmenopause	0.90 ± 0.11	0.85 ± 0.12	Wilcoxon	0	<0.001	0
Biochemical parameters							
HbA1C	Premenopause	5.47 ± 0.30	5.29 ± 0.32	Wilcoxon	1,421.5	<0.01	0.3
	Perimenopause	5.61 ± 0.53	5.19 ± 0.39	Paired *t*-test	7.46	<0.001	0.76
	Postmenopause	6.06 ± 0.34	5.70 ± 0.38	Wilcoxon	0	<0.001	0
Glucose	Premenopause	93.37 ± 10.53	87.84 ± 9.76	Paired *t*-test	3.84	<0.001	0.39
	Perimenopause	100.39 ± 5.64	95.22 ± 5.65	Paired *t*-test	6.14	<0.001	0.62
	Postmenopause	105.96 ± 7.76	98.00 ± 7.25	Paired *t*-test	7.62	<0.001	0.77
Hb	Premenopause	11.13 ± 2.39	11.21 ± 2.49	Wilcoxon	1,776	0.03	0.37
	Perimenopause	9.95 ± 1.84	10.54 ± 1.41	Wilcoxon	132	<0.001	0.03
	Postmenopause	10.46 ± 2.24	10.50 ± 1.56	Wilcoxon	1,866	0.7	0.39
Physical parameters							
SBP	Premenopause	113.54 ± 13.71	115.76 ± 12.61	Wilcoxon	1,064	<0.001	0.22
	Perimenopause	118.87 ± 11.56	117.27 ± 12.17	Paired *t*-test	0.97	0.33	0.1
	Postmenopause	123.75 ± 14.51	120.59 ± 15.14	Paired *t*-test	1.37	0.17	0.14
DBP	Premenopause	77.36 ± 5.84	75.21 ± 6.04	Paired *t*-test	2.57	0.01	0.26
	Perimenopause	78.64 ± 7.30	76.84 ± 6.53	Paired *t*-test	1.78	0.08	0.18
	Postmenopause	80.96 ± 6.72	78.26 ± 6.63	Paired *t*-test	2.9	<0.01	0.29
Body composition							
Skeletal Muscle Mass	Premenopause	66.71 ± 6.42	67.35 ± 4.40	Wilcoxon	1,608.5	<0.01	0.34
	Perimenopause	64.95 ± 5.13	67.72 ± 6.92	Wilcoxon	427.5	<0.001	0.09
	Postmenopause	64.48 ± 4.88	66.52 ± 5.37	Wilcoxon	97	<0.001	0.02
Fat mass	Premenopause	33.21 ± 6.59	32.62 ± 4.47	Wilcoxon	1,655.5	<0.01	0.35
	Perimenopause	35.26 ± 5.24	32.30 ± 6.95	Wilcoxon	345	<0.001	0.07
	Postmenopause	35.37 ± 5.13	33.50 ± 5.41	Wilcoxon	194	<0.001	0.04

Note: Mean ± SD, mean ± standard deviation; BMI, body mass index; WC, waist circumference; HC, hip circumference; WHR, waist-hip ratio; HbA1C, glycated hemoglobin; Hb, hemoglobin; SBP, systolic blood pressure; DBP, diastolic blood pressure; SMM, skeletal muscle mass. Effect sizes are reported as Cohen's d for paired *t*-test analyses and as rank biserial correlation (*r*) for Wilcoxon signed-rank test analyses.

In the Menopausal Rating Scale (MRS) domains, significant reductions were found across all groups for the Somato-vegetative domain (Table 3): premenopause (*Z* = 516.5, *p* < 0.001, *r* = 0.11), perimenopause (*Z* = 169.5, *p* < 0.001, *r* = 0.04), and postmenopause (*Z* = 505, *p* < 0.001, *r* = 0.11). Similarly, significant improvements were noted in the psychological domain: premenopause (*Z* = 188, *p* < 0.001, *r* = 0.04), perimenopause (*Z* = 0, *p* < 0.001, *r* = 0), and postmenopause (*Z* = 0, *p* < 0.001, *r* = 0). The Urogenital domain showed significant changes only in the perimenopause (*Z* = 295, *p* < 0.001, *r* = 0.06) and postmenopause groups (*Z* = 254, *p* < 0.01, *r* = 0.05). Total MRS scores also showed significant reductions across all groups: premenopause (*Z* = 558.5, *p* < 0.001, *r* = 0.12), perimenopause (*Z* = 0, *p* < 0.001, *r* = 0), and postmenopause [t(67) = 9.75, *p* < 0.001, *d* = 0.99].

**Table 3:** Comparison of menopausal rating scale and wellbeing across menopausal stages before and after the 12-week obesity educator program.

Outcome measure	Group	Pre (Mean ± SD)	Post (Mean ± SD)	Test	Statistic	*p*-value	Effect size
Menopausal rating scale							
Somato-vegetative domain	Premenopause	3.46 ± 1.64	2.89 ± 1.84	Wilcoxon	516.5	<0.001	0.11
	Perimenopause	8.15 ± 1.78	5.41 ± 1.95	Wilcoxon	169.5	<0.001	0.04
	Postmenopause	5.93 ± 2.43	4.81 ± 2.23	Wilcoxon	505	<0.001	0.11
Psychological domain	Premenopause	3.23 ± 1.67	2.75 ± 1.55	Wilcoxon	188	<0.001	0.04
	Perimenopause	7.27 ± 1.47	5.50 ± 1.65	Wilcoxon	0	<0.001	0
	Postmenopause	6.16 ± 1.71	5.01 ± 1.82	Wilcoxon	0	<0.001	0
Urogenital domain	Premenopause	1.09 ± 0.99	0.91 ± 0.85	Wilcoxon	454.5	0.1	0.1
	Perimenopause	2.19 ± 1.04	1.54 ± 1.03	Wilcoxon	295	<0.001	0.06
	Postmenopause	1.44 ± 1.01	1.21 ± 1.09	Wilcoxon	254	<0.01	0.05
Total MRS	Premenopause	7.78 ± 2.31	6.55 ± 2.77	Wilcoxon	558.5	<0.001	0.12
	Perimenopause	17.59 ± 2.45	12.32 ± 2.60	Wilcoxon	0	<0.001	0
	Postmenopause	13.54 ± 3.11	11.03 ± 3.53	Paired *t*-test	9.75	<0.001	0.99
Wellbeing							
Nutrition well-being (N-WB12)	Premenopause	5.96 ± 1.25	4.69 ± 1.41	Wilcoxon	168.5	<0.001	0.04
	Perimenopause	7.95 ± 2.31	5.59 ± 2.20	Wilcoxon	159.5	<0.001	0.03
	Postmenopause	6.12 ± 2.73	5.10 ± 2.20	Wilcoxon	0	<0.001	0
Energy	Premenopause	7.04 ± 1.50	7.12 ± 1.83	Wilcoxon	1,373.5	0.64	0.29
	Perimenopause	5.86 ± 1.65	6.87 ± 1.85	Wilcoxon	858	<0.001	0.18
	Postmenopause	6.72 ± 2.00	7.04 ± 2.12	Wilcoxon	578	0.03	0.12
Physical well-being (P-WB12)	Premenopause	8.13 ± 1.37	8.97 ± 1.10	Wilcoxon	635	<0.001	0.13
	Perimenopause	6.31 ± 1.24	6.96 ± 1.46	Wilcoxon	0	<0.001	0
	Postmenopause	7.05 ± 2.70	7.62 ± 1.76	Wilcoxon	717.5	<0.01	0.15
General well-being (GenWB)	Premenopause	21.22 ± 2.41	23.40 ± 2.32	Paired *t*-test	−7.07	<0.001	−0.72
	Perimenopause	16.22 ± 3.21	20.24 ± 3.14	Paired *t*-test	−9.25	<0.001	−0.94
	Postmenopause	19.65 ± 4.34	21.56 ± 3.75	Wilcoxon	670	<0.001	0.14

Note: MRS, menopausal rating scale; Mean ± SD, Mean ± standard deviation; N-WB12, negative well-being; P-WB12, positive well-being; GenWB, general well-being. Effect sizes are reported as Cohen's *d* for paired *t*-test analyses and as rank biserial correlation (*r*) for Wilcoxon signed-rank test analyses.

In terms of well-being, significant improvements were observed in Negative Well-being (N-WB12) across all groups (Table 3): premenopause (*Z* = 168.5, *p* < 0.001, *r* = 0.04), perimenopause (*Z* = 159.5, *p* < 0.001, *r* = 0.03), and postmenopause (*Z* = 0, *p* < 0.001, *r* = 0). For Energy, no significant change was observed in the premenopause group (*Z* = 1,373.5, *p* = 0.64), but significant improvements were noted in both the perimenopause (*Z* = 858, *p* < 0.001, *r* = 0.18) and postmenopause groups (*Z* = 578, *p* = 0.03, *r* = 0.12). Physical Well-being (P-WB12) showed significant improvements in all groups: premenopause (*Z* = 635, *p* < 0.001, *r* = 0.13), perimenopause (*Z* = 0, *p* < 0.001, *r* = 0), and postmenopause (*Z* = 717.5, *p* < 0.01, *r* = 0.15). General Well-being (GenWB) significantly increased across all groups: premenopause [t(67) = −7.07, *p* < 0.001, *d* = −0.72], perimenopause [t(67) = −9.25, *p* < 0.001, *d* = −0.94], and postmenopause (*Z* = 670, *p* < 0.001, *r* = 0.14).

ANOVA analyses assessed differences across menopausal groups for anthropometric, biochemical, and physical parameters (Table 4). Weight (*F* = 15.06, *p* < 0.001), BMI (*F* = 14.21, *p* < 0.001), WC (*F* = 13.28, *p* < 0.001), and WHR (*F* = 7.96, *p* < 0.001) showed significant group effects, with *post-hoc* Tukey tests confirming differences across all groups. HbA1C (*F* = 51.95, *p* < 0.001) and Glucose (*F* = 44.66, *p* < 0.001) also showed significant group effects, with *post-hoc* analyses revealing significant differences between perimenopause and premenopause, and postmenopause and premenopause (*p* < 0.001 for each). Hemoglobin (*F* = 4.34, *p* = 0.0139) and Systolic Blood Pressure (*F* = 3.31, *p* = 0.038) showed significant group effects, while Diastolic Blood Pressure (*F* = 5.51, *p* < 0.01) showed group differences.

**Table 4:** Analysis of variance (ANOVA) and *post-hoc* comparisons for anthropometric, biochemical, and physical parameters across menopausal stages.

Outcome Measure	F value	*p* value	Group Comparison	Post-hoc p-value
Height	2.87	0.0584	Perimenopause vs. Postmenopause	<0.001
			Perimenopause vs. Premenopause	<0.001
			Postmenopause vs. Premenopause	<0.001
Weight	15.06	<0.001	Perimenopause vs. Postmenopause	<0.001
			Perimenopause vs. Premenopause	<0.001
			Postmenopause vs. Premenopause	<0.001
BMI	14.21	<0.001	Perimenopause vs. Postmenopause	<0.001
			Perimenopause vs. Premenopause	<0.001
			Postmenopause vs. Premenopause	<0.001
WC	13.28	<0.001	Perimenopause vs. Postmenopause	0.8815
			Perimenopause vs. Premenopause	2.2784
			Postmenopause vs. Premenopause	<0.001
HC	16.85	<0.001	Perimenopause vs. Postmenopause	<0.001
			Perimenopause vs. Premenopause	2.2616
			Postmenopause vs. Premenopause	3.8595
WHR	7.96	<0.001	Perimenopause vs. Postmenopause	0.0198
			Perimenopause vs. Premenopause	<0.001
			Postmenopause vs. Premenopause	<0.001
HbA1C	51.95	<0.001	Perimenopause vs. Postmenopause	0.3815
			Perimenopause vs. Premenopause	<0.001
			Postmenopause vs. Premenopause	<0.001
Glucose	44.66	<0.001	Perimenopause vs. Postmenopause	0.1638
			Perimenopause vs. Premenopause	<0.001
			Postmenopause vs. Premenopause	<0.001
Hb	4.34	0.0139	Perimenopause vs. Postmenopause	<0.001
			Perimenopause vs. Premenopause	0.0288
			Postmenopause vs. Premenopause	0.0742
SBP	3.31	0.038	Perimenopause vs. Postmenopause	<0.001
			Perimenopause vs. Premenopause	<0.001
			Postmenopause vs. Premenopause	<0.001
DBP	5.51	<0.01	Perimenopause vs. Postmenopause	<0.001
			Perimenopause vs. Premenopause	<0.001
			Postmenopause vs. Premenopause	<0.001
SMM	1.14	0.3198	Perimenopause vs. Postmenopause	<0.001
			Perimenopause vs. Premenopause	<0.001
			Postmenopause vs. Premenopause	<0.001
Fat Mass	1.15	0.317	Perimenopause vs. Postmenopause	<0.001
			Perimenopause vs. Premenopause	<0.001
			Postmenopause vs. Premenopause	<0.001

Note: Mean ± SD, Mean ± standard deviation; BMI, body mass index; WC, waist circumference; HC, hip circumference; WHR; waist-hip ratio; HbA1C, glycated hemoglobin; Hb, hemoglobin; SBP, systolic blood pressure; DBP, diastolic blood pressure; SMM, skeletal muscle mass. *post-hoc p*-values are derived from Tukey's HSD test following one-way ANOVA to determine pairwise differences across menopausal stages.

In the Menopausal Rating Scale (MRS) domains (Table 5), the Somato-vegetative (*F* = 41.4, *p* < 0.001) and Psychological (*F* = 73.51, *p* < 0.001) domains showed significant group effects, with all *post-hoc* comparisons yielding significant differences. The Urogenital domain (*F* = 9.83, *p* < 0.001) and Total MRS score (*F* = 99.44, *p* < 0.001) revealed significant differences between groups. For Negative Well-being (N-WB12), a significant group effect was found (*F* = 5.03, *p* < 0.01). Energy did not show significant group differences (*F* = 0.45, *p* = 0.6392), while Physical Well-being (P-WB12) (*F* = 47.34, *p* < 0.001) and General Well-being (GenWB) (*F* = 25.08, *p* < 0.001) showed significant group differences.

**Table 5:** Analysis of variance (ANOVA) and *post-hoc* comparisons for menopausal symptoms and well-being across menopausal stages.

Variable	F value	*p* value	Group Comparison	***Post-hoc*** *p*-value
Somato-vegetative domain	41.4	<0.001	Perimenopause vs. Postmenopause	<0.001
			Perimenopause vs. Premenopause	<0.001
			Postmenopause vs. Premenopause	<0.001
Psychological domain	73.51	<0.001	Perimenopause vs. Postmenopause	<0.001
			Perimenopause vs. Premenopause	<0.001
			Postmenopause vs. Premenopause	<0.001
Urogenital domain	9.83	<0.001	Perimenopause vs. Postmenopause	<0.001
			Perimenopause vs. Premenopause	<0.001
			Postmenopause vs. Premenopause	<0.001
Total MS	99.44	<0.001	Perimenopause vs. Postmenopause	<0.001
			Perimenopause vs. Premenopause	<0.001
			Postmenopause vs. Premenopause	<0.001
N-WB12	5.03	<0.01	Perimenopause vs. Postmenopause	<0.001
			Perimenopause vs. Premenopause	<0.001
			Postmenopause vs. Premenopause	<0.001
Energy	0.45	0.6392	Perimenopause vs. Postmenopause	<0.001
			Perimenopause vs. Premenopause	<0.001
			Postmenopause vs. Premenopause	<0.001
P-WB12	47.34	<0.001	Perimenopause vs. Postmenopause	0.1636
			Perimenopause vs. Premenopause	1.5141
			Postmenopause vs. Premenopause	0.8543
GenWB	25.08	<0.001	Perimenopause vs. Postmenopause	0.262
			Perimenopause vs. Premenopause	2.1074
			Postmenopause vs. Premenopause	0.7878

Note: MS, menopausal symptoms; N-WB12, negative well-being; P-WB12, positive well-being; GenWB, general well-being; *post-hoc p*-values are derived from Tukey's HSD test following one-way ANOVA to determine pairwise differences across menopausal stages.

Correlation analyses depicted in Table 6 revealed that Weight was positively correlated with the psychological domain (*r* = 0.17, *p* < 0.01), Urogenital domain (*r* = 0.21, *p* < 0.001), Total MRS (*r* = 0.18, *p* < 0.01), and Negative Well-being (N-WB12) (*r* = 0.13, *p* < 0.05). Weight was negatively correlated with Physical Well-being (*r* = −0.23, *p* < 0.001) and General Well-being (*r* = −0.18, *p* < 0.01). WC was negatively correlated with the Somato-vegetative domain (*r* = −0.30, *p* < 0.001), Psychological domain (*r* = −0.39, *p* < 0.001), and Urogenital domain (*r* = −0.13, *p* < 0.05), but positively correlated with Physical Well-being (*r* = 0.32, *p* < 0.001) and General Well-being (*r* = 0.19, *p* < 0.001). WHR was negatively correlated with Somato-vegetative (*r* = −0.31, *p* < 0.001), Psychological (*r* = −0.39, *p* < 0.001), and Urogenital domains (*r* = −0.19, *p* < 0.01), and Total MRS (*r* = −0.43, *p* < 0.001), but positively correlated with Physical Well-being (*r* = 0.36, *p* < 0.001) and General Well-being (*r* = 0.26, *p* < 0.01). Fat Mass was negatively correlated with the psychological domain (*r* = −0.16, *p* < 0.01) and Total MRS (*r* = −0.17, *p* < 0.01), while positively correlated with Physical Well-being (*r* = 0.14, *p* < 0.05).

**Table 6:** Correlation between anthropometric, biochemical, and physical variables, menopausal symptoms, and well-being across menopausal groups.

Variable	Somato-vegetative	Psychological	Urogenital	Total MRS	Nutritional WB	Energy	Physical WB	General WB
Height	0.14 **	0.1	0.06	0.15 **	0.02	−0.06	−0.03	−0.06
Weight	0.07	0.17 **	0.21 ***	0.18 **	0.13 *	0.02	−0.23 ***	−0.18 **
BMI	−0.1	−0.05	0.02	−0.08	0.01	−0.01	−0.07	−0.05
WC	−0.30 ***	−0.39 ***	−0.13 *	−0.42 ***	−0.04	0.02	0.32 ***	0.19 ***
HC	−0.17 **	−0.23 ***	0	−0.23 ***	0.02	−0.02	0.13 *	0.04
WHR	−0.31 ***	−0.39 ***	−0.19 **	−0.43 ***	−0.08	0.06	0.36 ***	0.26 ***
SMM	0.09	0.16 **	0.09	0.16 **	−0.01	0	−0.13 *	−0.06
Fat Mass	−0.11	−0.16 **	−0.08	−0.17 **	0.03	0.01	0.14 *	0.06

Note: BMI, body mass index; WC, waist circumference; HC, hip circumference; WHR, waist-hip ratio; HbA1C, glycated hemoglobin; Hb, hemoglobin; SBP, systolic blood pressure; DBP, diastolic blood pressure; SMM, skeletal muscle mass. *p < 0.05. **p < 0.01. ***pz < 0.001.

Multiple regression analyses (Table 7) indicated that the model for the Somato-vegetative domain explained 15% of the variance (R-squared = 0.15), with Hemoglobin (*p* = 0.0248) as a significant predictor. The Psychological domain model explained 24% of the variance (R-squared = 0.24), with Waist-to-Hip Ratio (WHR) as a significant predictor (*p* = 0.0322). The Urogenital domain model explained 8% of the variance (R-squared = 0.08), with Weight (*p* = 0.0122) as the only significant predictor. For Total MRS, 27% of the variance was explained (R-squared = 0.27), with Height (*p* = 0.0214) and Waist-to-Hip Ratio (WHR) (*p* = 0.0266) as significant predictors. In terms of well-being, the model for Negative Well-being (N-WB12) explained 4% of the variance (R-squared = 0.04), with Weight (*p* = 0.0405) as a significant predictor. For Energy, the model explained 6% of the variance (R-squared = 0.06), with Height (*p* = 0.0177), BMI (*p* = 0.0217), and Glucose (*p* = 0.0105) as significant predictors. No significant predictors were identified for Positive Well-being (P-WB12) or General Well-being (GenWB).

**Table 7:** Regression analysis of menopausal symptoms and well-being with anthropometric and biochemical predictors.

Dependent Variable	Predictor Variable	B (Coefficient)	*p*-value	R-squared
MS somatovegetative domain	Height	8.5	0.0207	0.15
	Hb	0.45	0.0248	
MS psychological domain	WHR	−39.51	0.0322	0.24
MS urogenital domain	Weight	0.13	0.0122	0.08
Total MS	Height	13.37	0.0214	0.27
	WHR	−75.5	0.0266	
N-WB12	Weight	0.21	0.0405	0.04
Energy	Height	−7.78	0.0177	0.06
	BMI	−0.22	0.0217	
P-WB12	Glucose	0.02	0.0506	0.2
GenWB	None	–	–	0.11

Note: MS, menopausal symptoms; N-WB12, negative well-being; P-WB12, positive well-being; GenWB, general well-being.

## 4 Discussion

The findings of this study on the 12-week Obesity Educator Program align with existing research, demonstrating significant improvements in weight, BMI, and waist circumference across all menopausal stages, with postmenopausal women showing the most notable changes. This outcome is supported by studies reporting greater weight loss and BMI reductions in postmenopausal women following lifestyle interventions involving nutrition education and exercise (10, 14). The hormonal and physiological changes in postmenopausal women, particularly reduced estrogen levels, are linked to increased abdominal obesity and metabolic dysregulation, which heightens their responsiveness to weight management interventions (24, 25). Tailored lifestyle programs that adjust for individual factors, such as adherence and comorbidities, have been shown to enhance weight loss in both peri- and postmenopausal women (13).

The study also observed significant reductions in waist and hip circumferences, with postmenopausal women showing the largest decreases in waist circumference, addressing central obesity, a known risk factor for cardiovascular and metabolic diseases (26, 27). These results underscore the heightened risk of central obesity during postmenopause, contributing to complications like cardiovascular disease and type 2 diabetes (28, 29). Additionally, the program led to reductions in systolic and diastolic blood pressure, particularly in postmenopausal women, reflecting a lower risk of hypertension-related complications (30, 31).

The improvements in glucose and HbA1c levels, especially in postmenopausal women, align with previous findings from interventions focused on dietary modifications and glycemic control (32, 33). Hemoglobin levels also showed a slight increase, indicative of improved blood health across all groups. Increases in skeletal muscle mass (SMM) were observed in all groups, with perimenopausal women showing the greatest gains. This is crucial as muscle loss is a common issue during menopause, exacerbated by hormonal shifts (34, 35). Emerging evidence also suggests that the myokine irisin—induced through exercise and regulated by Fibronectin Type III Domain Containing 5 (FNDC5)—plays a significant role in mitigating postmenopausal metabolic syndrome by improving energy expenditure, skeletal muscle health, and lipid metabolism independent of estradiol (36). Fat mass changes were mixed, with increases noted in some perimenopausal participants, reflecting variability in fat redistribution, which may be influenced by intervention components and adherence (11, 12, 37).

The Menopausal Rating Scale (MRS) revealed significant improvements in somato-vegetative, psychological, and urogenital domains, particularly in perimenopausal women, who typically experience more severe symptoms due to hormonal fluctuations. These findings align with studies that have shown perimenopausal women to be more susceptible to psychological symptoms like anxiety and mood disturbances, which can be effectively managed through lifestyle interventions (38–40). The improvements in urogenital symptoms, especially in peri- and postmenopausal women, further support the program's effectiveness in addressing hormonal changes affecting sexual function and bladder health (15, 41). In line with these findings, the Endocrine Society Clinical Practice Guideline emphasizes the importance of lifestyle modifications for all postmenopausal women, while recommending low-dose vaginal estrogen, ospemifene, or non-hormonal therapies like vaginal lubricants and moisturizers to alleviate genitourinary symptoms (42).

Well-being, assessed through the W-BQ12, also improved significantly across all groups, particularly in negative well-being (N-WB12) and physical well-being (P-WB12), with perimenopausal women showing the greatest improvements. These changes are consistent with research demonstrating the benefits of educational interventions on psychological well-being during menopause (15, 43). The improvements in energy and reductions in negative well-being suggest that the program effectively addressed the psychological and physical challenges unique to this transitional phase.

Several studies emphasize the importance of incorporating behavior change techniques, psychological support, and emerging technologies, such as mobile health applications and wearable devices, into menopausal health interventions (44–46). Previous study have also, confirmed that menopausal symptoms significantly impair work ability, increase absenteeism, and reduce overall productivity (47). These results reinforce the necessity of workplace-level interventions and employer-supported strategies tailored to women undergoing menopausal transition. These innovations offer personalized interventions and real-time monitoring, potentially enhancing long-term adherence and health outcomes. Overall, the 12-week Obesity Educator Program was effective in improving anthropometric, biochemical, and psychological outcomes across all stages of menopause. By tailoring interventions to the specific needs of women at different menopausal stages, this program has shown potential for long-term health benefits, emphasizing the importance of personalized and stage-specific strategies in menopausal health management.

## 5 Practical implications

The findings of this study have important practical implications for healthcare providers and policymakers. The observed reductions in weight, BMI, and waist-to-hip ratio across all menopausal groups suggest that weight management programs tailored to menopausal women can play a critical role in improving both physical and psychological health. Such programs may help alleviate somatic symptoms, such as fatigue and body pain, as well as psychological issues like mood swings and anxiety. Additionally, the identification of significant predictors, such as waist-to-hip ratio and hemoglobin levels, highlights the need for personalized health interventions that focus on both metabolic and psychological factors. These insights can guide the development of more effective, stage-specific interventions for menopausal women, aimed at enhancing their overall well-being and quality of life. Furthermore, healthcare providers can use this information to better educate and counsel women on managing the physical and psychological challenges of menopause, particularly in culturally sensitive contexts like India, where discussions around menopause are often stigmatized.

## 6 Limitations of this study

This study has a few limitations that should be acknowledged. First, the cross-sectional design does not allow for the establishment of causality between menopausal symptoms, psychological well-being, and the identified predictors. Longitudinal studies would be necessary to explore how these factors interact over time. Additionally, the study population was limited to obese women in the Delhi NCR region, which may not fully represent the diverse experiences of menopausal women across different regions of India or other cultural backgrounds. While BMI and HbA1c were used as proxy indicators of metabolic and hormonal status, direct hormonal assessments such as serum estrogen levels could not be conducted due to resource constraints and socioeconomic limitations among participants. The reliance on self-reported measures, such as the Menopause Rating Scale (MRS) and Well-being Questionnaire (W-BQ12), could introduce reporting bias, as participants may underreport or over report symptoms due to social or personal factors. Furthermore, body composition was assessed using bioelectrical impedance analysis (BIA), which does not differentiate regional fat compartments (e.g., trunk, android, or gynecoid fat) that may have distinct physiological relevance. Additionally, adipokines such as leptin and adiponectin were not measured, which could have provided further insight into fat metabolism and hormonal interactions. Future research should address these limitations by incorporating a more diverse population, using longitudinal designs, integrating hormonal and adipokine profiling, and employing more advanced body composition techniques such as dual-energy x-ray absorptiometry (DEXA).

## 7 Conclusion

This study underscores the positive impact of a 12-week obesity educator program on alleviating menopausal symptoms and enhancing well-being across different stages of menopause. The program led to improvements in body composition, including reductions in weight, BMI, and waist-to-hip ratio, as well as better glycemic control. Moreover, the reduction in somatic and psychological symptoms, particularly in perimenopausal and postmenopausal women, highlights the program's effectiveness in addressing the multifaceted challenges of menopause. Key predictors such as hemoglobin levels, weight, and waist-to-hip ratio emerged as significant factors influencing symptom severity and well-being. These insights emphasize the importance of comprehensive health strategies that target both physical and psychological aspects of menopause, ultimately contributing to improved health outcomes and quality of life for women undergoing this transition.

## Data Availability

The raw data supporting the conclusions of this article will be made available by the authors, without undue reservation.
